# Patterns of Human Milk Oligosaccharides in Mature Milk Are Associated with Certain Gut Microbiota in Infants

**DOI:** 10.3390/nu16091287

**Published:** 2024-04-25

**Authors:** Shuai Mao, Ai Zhao, Hua Jiang, Jingyu Yan, Wuxian Zhong, Yiping Xun, Yumei Zhang

**Affiliations:** 1 Department of Nutrition and Food Hygiene, School of Public Health, Peking University, Beijing 100191, China; maoshui@pku.edu.cn (S.M.); zhongwuxian@pku.edu.cn (W.Z.); 2 Vanke School of Public Health, Tsinghua University, Beijing 100084, China; aizhao18@tsinghua.edu.cn; 3 School of Nursing, Peking University, Beijing 100091, China; jianghua@bjmu.edu.cn; 4 CAS Key Laboratory of Separation Science for Analytical Chemistry, Dalian Institute of Chemical Physics, Chinese Academy of Sciences, Dalian 116023, China; yanjingyu@dicp.ac.cn; 5 Junlebao Dairy Joint Laboratory of Breast Milk Science and Life Health, Peking University, Beijing 100191, China; xunyiping@jlbry.com

**Keywords:** human milk oligosaccharides, mature milk HMO patterns, secretor phenotype, infant gut microbiome

## Abstract

Human milk oligosaccharides (HMOs) are complexes that play a crucial role in shaping the early-life gut microbiota. This study intends to explore whether HMO patterns are associated with the gut microbiota of infants. We included 96 Chinese breastfeeding mother–infant dyads. Breast milk and infant faecal samples were collected and tested. With milk 2′-fucosyllactose, difucosyllactose, and lacto-*N*-fucopentaose-I as biomarkers, we divided the mothers into secretor and non-secretor groups. HMO patterns were extracted using principal component analysis. The majority (70.7%) of mothers were categorised as secretor and five different HMO patterns were identified. After adjustment, the infants of secretor mothers exhibited a lower relative abundance of *Bifidobacterium bifidum* (β = −0.245, 95%CI: −0.465~−0.025). An HMO pattern characterised by high levels of 3-fucosyllactose, lacto-*N*-fucopentaose-III, and lacto-*N*-neodifucohexaose-II was positively associated with the relative abundance of *Bifidobacterium breve* (*p* = 0.014), while the pattern characterised by lacto-*N*-neotetraose, 6′-sialyllactose, and sialyllacto-*N*-tetraose-b was negatively associated with *Bifidobacterium breve* (*p* = 0.027). The pattern characterised by high levels of monofucosyl-lacto-*N*-hexaose-III and monofucosyl-lacto-*N*-neohexaose was positively associated with *Bifidobacterium dentium* (*p* = 0.025) and *Bifidobacterium bifidum* (*p* < 0.001), respectively. This study suggests that HMO patterns from mature breast milk were associated with certain gut microbiota of breastfed infants.

## 1. Introduction

The World Health Organization (WHO) recommends commencing breastfeeding within 1 h of birth and maintaining exclusive breastfeeding until the baby reaches 6 months of age [1]. Breast milk is the most suitable food for infants aged 0–6 months, as it not only provides infants with the necessary nutrients for their growth and development, but also contains human milk oligosaccharides (HMOs), hormones, cytokines, and other bioactive substances [2,3,4,5]. The nutrients and bioactive substances in breast milk have different patterns of characteristics in colostrum, transitional milk, and mature milk, thus meeting the needs of infants [6,7,8].

The collective term HMOs refers to a class of covalently linked, multifunctional, indigestible, and structurally diverse polysaccharides unique to human breast milk. They are the third largest solid component in breast milk, following lactose and fat [9,10]. Although researchers had discovered as early as the beginning of 20th century that human milk contains unique carbohydrates that can promote the growth of *Bifidobacterium bifidum* (*B. bifidum*), the exact structure and other potential functions of these carbohydrates were difficult to determine due to limitations in detection techniques [11]. With the development of analytical technology, more than 200 types of HMOs have been identified [12] and the structure, function, and application of HMOs have become research hotspots in recent years.

The concentration of HMOs is influenced by multiple genetic and non-genetic factors. Genetically, the secretor gene has been reported to be one of the most crucial determinants of HMOs [13]. The secretor gene encodes fucosyltransferase 2 (FUT2), which mediates the synthesis of α-1,2 glycosidic bonded HMOs [14]. Thus, the absence of FUT2 results in an HMO pattern characterised by extremely low levels or an absence of α-1,2 glycosidic bonded HMOs such as 2′-fucosyllactose (2′-FL), difucosyllactose (DFL), and lacto-*N*-fucopentaose I (LNFP-I) in non-secretor (nSe) mothers. Reports on the percentage of secretor (Se) in Chinese mothers ranges from 50.0% to 81.2% [15,16,17,18], while reviews covering populations from other countries indicated that the percentage of Se is about 80% [19,20]. In addition, non-genetic factors including maternal age, the stage of milk maturation, race, geographic location, and parity also have an impact on HMO concentration [16,21,22,23,24]. Such differences may suggest a unique distribution of HMO patterns and, subsequently, different infant gut microbiota composition in the Chinese population.

However, current studies regarding the association of HMOs with infant gut microbiota in China are mostly focused on the individual HMOs, whereas the HMO patterns are still yet to be considered. A study conducted in Jining and Harbin reported that the concentrations of DFL and LNFP-I in Jining mothers were higher than in Harbin mothers, and the relative abundance of *Bifidobacterium* in the gut of Jining infants was consistently higher than that of Harbin infants [25]. It is of interest that both DFL and LNFP-I were FUT2-related HMOs that predominantly appear in the Se milk [26]. As the maternal secretor phenotype could shape the HMOs patterns, Bai et al. [15] followed up on mother–infant dyads in Jinzhou and observed that, in contrast to infants fed by Se mothers, those fed by nSe mothers displayed a more substantial shift in the composition of their gut microbiota during lactation. Additionally, the abundance of *Bifidobacterium breve* (*B. breve*) was higher in this group. However, there have also been studies suggested that the maternal secretor phenotype is not associated with infant gut microbiota composition [27,28]. These contradictory findings regarding the association of HMOs and the maternal secretor phenotype with infant gut microbiota connotes that a priori Se/nSe HMO patterns might not be sufficient enough in emphasising how the HMOs interacts with infant gut microbiota as a complex.

Using paired maternal–infant data, this study intends to explore the association of different levels and patterns of HMOs in mature milk with the composition of infant gut microbiota.

## 2. Materials and Methods

### 2.1. Study Population

This study is part of the 13th Five Year Plan for the National Key Research and Development Program of China (2017YFD0400602). Mother-infant dyads (*n* = 96) from Chengdu (*n* = 15), Guangzhou (*n* = 10), Hohhot (*n* = 14), Beijing (*n* = 39), and Suzhou (*n* = 19), representing western, southern, northern, and eastern China, were recruited for this study.

Mothers who were healthy and aged 20–45 years were considered eligible for inclusion if they had (1) no alcohol or tobacco use; (2) a full-term delivery (37–42 weeks); (3) given birth 1–3 months ago; and (4) exclusively breastfed. Mothers who had (1) mastitis or gastrointestinal infections; (2) used probiotics in the last 3 months; or (3) no milk sample were excluded. Corresponding to their mothers, infants with faecal samples were included, while those with (1) gastrointestinal infections, (2) limb disabilities, or (3) a history of probiotic or prebiotic use after birth were excluded.

### 2.2. Basic Information Collection

Trained investigators used interview questionnaires to collect the information required about the subjects, including residential city (Chengdu, Guangzhou, Hohhot, Suzhou, Beijing), maternal age (years), infant age (days), delivery modes (caesarean or vaginal), and parity (primipara or multipara).

### 2.3. Sample Collection and Preservation

The breast milk sample was collected following the previously established standard process [16]. On the day before the investigation, the investigator contacted the subjects by phone and instructed the mothers to complete breastfeeding and empty their breasts before 7 a.m. on the day of the investigation. Breast milk samples were collected between 9 and 11 a.m. in the morning to avoid the influence of circadian rhythms. Trained investigators helped the maternal subjects to collect a total of 45 mL whole milk (including fore and hind milk) into a sterile tube. The remaining milk was returned to the mother and the milk sample was gently mixed and then immediately stored at –80 °C until testing.

An infant faecal sample was collected using a sterilised plastic scoop attached to the inner aspect of the lid of a sterile polypropylene vial with a DNA preservation solution. Parents were asked to collect 0.5 g of infant faecal sample (approximately the size of one peanut) at home around the time of breast milk collection (±24 h) and store the tube at −20 °C immediately after the collection. Samples were transported to the local research institution the next day via ice bags and insulation bags, then stored at −80 °C until testing.

### 2.4. HMO Analysis

Before analysis, the samples were defatted and diluted. An ACQUITY UPLC I-Class system (Waters, Milford, MA, USA) coupled to a Xevo TQ-XS triple quadrupole mass spectrometer (Waters) was used for analysis. Chromatography was performed on a Waters ACQUITY BEH Amide column (130 Å, 1.7 μm, 2.1 × 150 mm). ESI-MS detection was in the negative-ion mode and collision-induced dissociation tandem MS (CID-MS/MS) was carried out using multi-reaction monitoring (MRM) for both sequence assignment and quantitation. The details of this method have been described in a previous study [29].

Twenty-three HMOs were detected in this study, including 2′-FL, 3-fucosyllactose (3-FL), DFL, LNFP-I, lacto-*N*-fucopentaose II (LNFP-II), lacto-*N*-fucopentaose III (LNFP-III), lacto-*N*-difucohexaose I (LNDFH-I), lacto-*N*-difucohexaose II (LNDFH-II), lacto-*N*-neodifucohexaose I (LNnDFH-I), lacto-*N*-neodifucohexaose II (LNnDFH-II), monofucosyl-lacto-*N*-hexaose I (MFLNH-I), monofucosyl-lacto-*N*-hexaose III (MFLNH-III), monofucosyl-lacto-*N*-neohexaose (MFLNnH), difucosyl-para-lacto-*N*-neohexaose (DFpLNnH), lacto-*N*-tetraose (LNT), lacto-*N*-neotetraose (LNnT), 3′-sialyllactose (3′-SL), 6′-sialyllactose (6′-SL), sialyllacto-*N*-tetraose b (LSTb), sialyllacto-*N*-tetraose c (LSTc), disialyllacto-*N*-tetraose (DSLNT), 3-sialyl-latco-*N*-fucopentaose II (3′-SLNFP II), and 6-sialyl-latco-*N*-fucopentaose VI (6′-SLNFP VI). Additionally, the concentration of 3′-SLNFP-II and 6′-SLNFP-VI was presented as 3′-SLNFP-II&6′-SLNFP-VI.

The maternal secretor phenotype was determined based on the presence of 2′-FL, DFL, and LNFP-I [26]. Mothers with high levels of 2′-FL, DFL, and LNFP-I were classified as Se, while those with 2′-FL, DFL, and LNFP-I below the limit of detection (LOD) were classified as nSe.

### 2.5. 16SRNA Sequencing

16SRNA sequencing was conducted by a 16S full-length polymerase chain reaction (PCR) amplification system provided by Biomarker Technologies Co., Ltd. (Beijing, China). The sequencing was performed as described in previous studies [30]. Briefly, the DNA was extracted from 96 faecal samples and examined for quality, quantity, concentration, and purity. Then, primer pairs 27F (AGRGTTTGATYNTGGCTCAG) and 1492R (TASGGHTACCTTGTTASGACTT) were used for PCR amplification. The PCR amplicons were subsequently purified and quantified. Libraries were prepared from the equally pooled individual DNA amplicons and the purified libraries were sequenced on a PacBio Sequel II platform (Beijing Biomarker Technologies Co., Ltd., Beijing, China)

Circular consensus sequencing (CCS) reads were generated from the faecal samples by barcode-based identification. Full-length amplicon tags were generated from the effective CCS reads which were filtered, clustered, and denoised. Usearch (version 10.0) was used to establish operational taxonomic unit (OTU) by clustering reads with a similarity threshold at 97.0% [31]. With SILVA (Release132, https://www.arb-silva.de/documentation/release-132/ (accessed on 13 December 2017)) as a reference database, the naive Bayesian classifier algorithm was used to obtain species classification information. Infant gut microbiota composition at different taxonomic levels was then calculated.

### 2.6. Statistical Analysis

R software (version 4.3.2) was used to analyse the data. All statistical tests were two-sided, with *p* < 0.05 indicating statistically significant differences.

The Shapiro–Wilk method was used to confirm whether the distribution of variables obeyed normality. Results were presented as mean ± standard deviation (SD) for normally distributed continuous data, and median (P25, P75) for continuous non-normal ones. Categorical variables were summarised as numbers and percentages (*n*, %). Student’s *t*-tests, analysis of variance (ANOVA), or Kruskall–Wallis tests were used for continuous normal or non-normal variables, and the chi-squared test was applied for the categorical variables.

Principal component analysis (PCA) was performed to draft the patterns of HMOs with a varimax-rotation method. PCA pattern scores were then calculated accordingly. We selected 0.5 as the threshold for the absolute value of factor loading. Thus, certain HMOs with a factor loading of ≥0.5 or ≤−0.5 would be taken as representative HMOs for each pattern.

The association of the relative abundance of infant gut microbiota with maternal secretor phenotypes and HMO patterns was explored using general linear regression and was adjusted for cities (Chengdu, Guangzhou, Hohhot, Beijing, Suzhou), maternal age (≤30 y or >30 y), total concentration of HMOs (mg/L), and mode of delivery (vaginal delivery or caesarean section). And to improve normality of the residuals, the relative abundance of infant gut microbiota was transformed in the form of lg (X + 1).

### 2.7. Ethical Considerations

This study was approved by the Medical Ethics Research Board of Peking University (No. IRB00001052-19040) and complied with the Declaration of Helsinki. Written informed consent was obtained from all participants.

## 3. Results

### 3.1. Characteristics of the Studied Population

In this study, 96 mother–infant dyads were included, among which 42 mothers (43.8%) were aged 30 years or below when they delivered. There were 29 mothers who delivered by caesarean section (30.2%), and 71 mothers were primiparous (74.0%).

### 3.2. HMO Concentration

The concentration of 14 neutral fucosylated HMOs (2′-FL, 3-FL, DFL, LNFP-I, LNFP-II, LNFP-III, LNDFH-I, LNDFH-II, LNnDFH-I, LNnDFH-II, MFLNH-I, MFLNH-III, MFLNnH, and DFpLNnH), 2 neutral nonfucosylated HMOs (LNT, LNnT), and 7 sialylated HMOs (3′-SL, 6′-SL, LSTb, LSTc, DSLNT, 3′-SLNFP-II, and 6′-SLNFP-VI) were detected.

Among 96 maternal subjects included, 70 (72.9%) of them were identified as Se. As shown in Table 1, for the 23 HMOs detected, the total HMO concentration in nSe mothers was slightly higher than in the Se mothers, but this difference was not significant.

There were significant differences in the total concentration of HMOs between cities. There was no significant difference in the distribution of total HMOs among participants in different maternal age groups, with different delivery modes, or parities.

In the 96 milk samples we detected, the average of total concentration of HMOs was 6109.2 mg/L. 2′-FL was the most abundant HMO, with a median concentration of 1514.0 mg/L. 3-FL (583.0 mg/L) was the second most abundant neutral fucosylated HMO, while LNT (458.7 mg/L) and 6′-SL (253.9 mg/L) were the most abundant neutral nonfucosylated HMOs and sialylated HMOs, respectively. Certain HMO concentrations also varied with different maternal and infant characteristics (data shown in Appendix A). There were maternal subjects with HMO concentrations under the LOD, including 26 subjects (27.1%) with 2′-FL under the LOD, 25 subjects (26.0%) with DFL, 25 subjects (26.0%) with LNFP-I, 8 subjects (8.3%) with LNFP-II, 19 subjects (19.8%) with LNDFH-I, 38 subjects (39.6%) with LNDFH-II, 26 subjects (27.1%) with LNnDFH-I, 5 subjects (5.2%) with LNnDFH-II, 32 subjects (33.3%) with MFLNH-I, 2 subjects (2.1%) with LNnT, and 2 subjects (2.1%) with LSTb.

### 3.3. Infant Faecal Microbiota

At the phylum level, the infant gut microbiota was dominated by *Firmicutes* (39.5%), *Proteobacteria* (36.5%), *Bacteroidota* (13.6%), and *Actinobacteriota* (9.9%). At the genus level, *Clostridium sensu stricto 1* (*C. sensu stricto 1*, 18.4%), *Klebsiella* (15.7%), *Escherichia Shigella* (10.8%), *Bacteroides* (10.6%), and *Bifidobacterium* (9.2%) were the dominant genera. At the species level, the infant gut microbiota was dominated by *Klebsiella pneumoniae* (*K. pneumoniae*, 12.2%), *Escherichia coli* (*E. coli*, 10.6%), *Clostridium perfringens* (*C. perfringens*, 8.1%), *Bifidobacterium longum* (5.4%), and *Bacteroides fragilis* (4.8%).

### 3.4. HMOs and the Gut Microbiome of Breastfed Infants

Firstly, we explored the association between maternal secretor phenotype and the eight most abundant infant gut *Bifidobacterium* and *Lactobacilli* (Table 2).

After adjusting for city, maternal age, total concentration of HMOs, and delivery mode, the infants who were nourished by Se mothers were demonstrated to have a lower relative abundance of *B. bifidum*. The association between maternal secretor phenotype and the 15 most abundant infant gut microbiota except for *Bifidobacterium* and *Lactobacilli* were also explored (Supplemental Table S1). After adjustment, the infants who were nourished by Se mothers exhibited a lower relative abundance of *Parabacteroides distasonis* (*P. distasonis*). 

**Figure 1:**
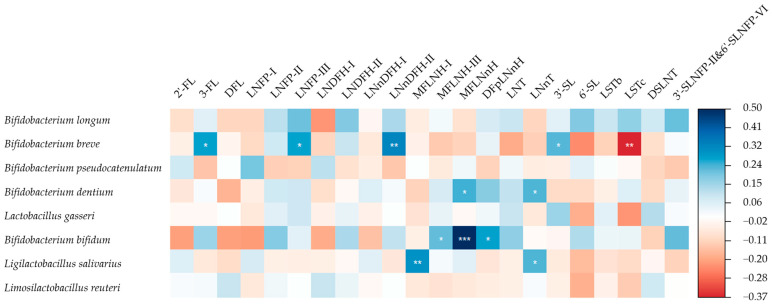
Standardised coefficients between individual HMO concentrations and relative abundance of *Bifidobacterium* and *Lactobacilli*. *: *p* < 0.05, **: *p* < 0.01, ***: *p* < 0.001. The eight most abundant infant gut microbiota from *Bifidobacterium* and *Lactobacilli* were explored. Adjusted for city, maternal age, total concentration of HMOs, and delivery mode. The relative abundance of infant gut microbiota was transformed in the form of lg (X + 1). 2′-FL: 2′-fucosyllactose, 3-FL: 3-fucosyllactose, DFL: difucosyllactose, LNFP-I: lacto-*N*-fucopentaose I, LNFP-II: lacto-*N*-fucopentaose II, LNFP-III: lacto-*N*-fucopentaose III, LNDFH-I: lacto-*N*-difucohexaose I, LNDFH-II: lacto-*N*-difucohexaose II, LNnDFH-I: lacto-*N*-neodifucohexaose I, LNnDFH-II: lacto-*N*-neodifucohexaose II, MFLNH-I: monofu-cosyl-lacto-*N*-hexaose I, MFLNH-III: monofucosyl-lacto-*N*-hexaose III, MFLNnH: monofucosyl-lacto-*N*-neohexaose, DFpLNnH: difucosyl-para-lacto-*N*-neohexaose, LNT: lac-to-*N*-tetraose, LNnT: lacto-*N*-neotetraose, 3′-SL: 3′-sialyllactose, 6′-SL: 6′-sialyllactose, LSTb: sialyllacto-*N*-tetraose b, LSTc: sialyllacto-*N*-tetraose c, DSLNT: disialyllac-to-*N*-tetraose, 3′-SLNFP-II: 3-sialyl-latco-*N*-fucopentaose II, 6′-SLNFP-VI: 6-sialyl-latco-*N*-fucopentaose VI, Sum: total concentration of the HMOs detected.

The association between individual HMO concentrations and infant gut microbiota was then explored. As shown in Figure 1, when city, maternal age, total concentration of HMOs, and delivery mode were controlled for, the relative abundance of *B. breve* was positively associated with the concentrations of numerous HMOs including 3-FL, LNFP-III, LNnDFH-II, and 3′-SL, while it was negatively associated with LSTc. The relative abundance of *Bifidobacterium dentium* (*B. dentium*) was positively associated with the concentrations of MFLNnH and LNnT. The relative abundance of *B. bifidum* was positively associated with the concentrations of MFLNH-III, MFLNnH, and DFpLNnH. The relative abundance of *Ligilactobacillus salivarius* (*Lgb. salivarius*) was positively associated with the concentrations of MFLNH-I and LNnT. The result of the crude model is shown in Supplemental Figure S1.

**Figure 2:**
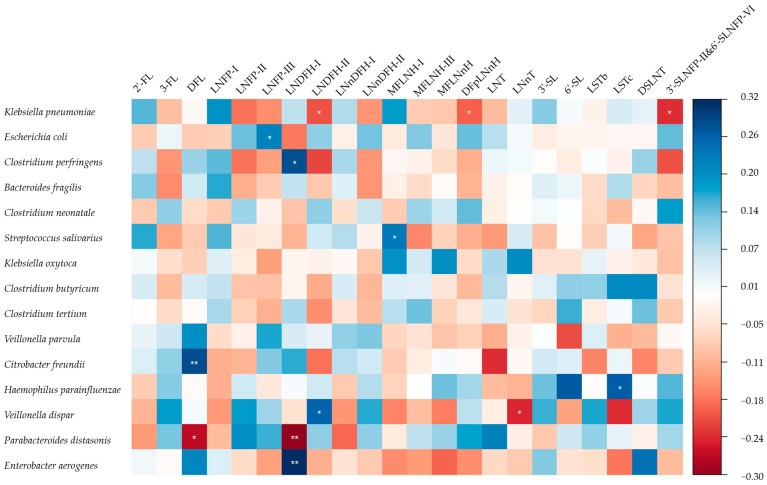
Standardised coefficients between individual HMO concentrations and the relative abundance of commensal bacteria. *: *p* < 0.05, **: *p* < 0.01. The 15 most abundant infant gut microbiota except for *Bifidobacterium* and *Lactobacilli* were explored. Adjusted for city, maternal age, total concentration of HMOs, and deliver mode. The relative abundance of infant gut microbiota was transformed in the form of lg (X + 1). 2′-FL: 2′-fucosyllactose, 3-FL: 3-fucosyllactose, DFL: difucosyllactose, LNFP-I: lacto-*N*-fucopentaose I, LNFP-II: lacto-*N*-fucopentaose II, LNFP-III: lacto-*N*-fucopentaose III, LNDFH-I: lacto-*N*-difucohexaose I, LNDFH-II: lacto-*N*-difucohexaose II, LNnDFH-I: lacto-*N*-neodifucohexaose I, LNnDFH-II: lacto-*N*-neodifucohexaose II, MFLNH-I: monofu-cosyl-lacto-*N*-hexaose I, MFLNH-III: monofucosyl-lacto-*N*-hexaose III, MFLNnH: monofucosyl-lacto-*N*-neohexaose, DFpLNnH: difucosyl-para-lacto-*N*-neohexaose, LNT: lac-to-*N*-tetraose, LNnT: lacto-*N*-neotetraose, 3′-SL: 3′-sialyllactose, 6′-SL: 6′-sialyllactose, LSTb: sialyllacto-*N*-tetraose b, LSTc: sialyllacto-*N*-tetraose c, DSLNT: disialyllac-to-*N*-tetraose, 3′-SLNFP-II: 3-sialyl-latco-*N*-fucopentaose II, 6′-SLNFP-VI: 6-sialyl-latco-*N*-fucopentaose VI, Sum: total concentration of the HMOs detected.

The adjusted association between HMO concentrations and the 15 most common commensal bacteria in the infant gut was also explored and is shown in Figure 2. After adjustment, the relative abundance of *E*. *coli, C. perfringens*, *Streptococcus salivarius* (*S. salivarius*), *Citrobacter freundii* (*C. freundii*), *Haemophilus parainfluenzae* (*H. parainfluenzae*), *Veillonella dispar* (*V. dispar*), and *Enterobacter aerogenes* (*E. aerogenes*) were positively associated with the concentrations of LNFP-III, LNDFH-I, MFLNH-I, DFL, LSTc, LNDFH-II, and LNDFH-I, respectively. Meanwhile, the relative abundance of *K. pneumoniae* was negatively associated with the concentrations of LNDFH-II, DFpLNnH, and 3′-SLNFP-II&6′-SLNFP-VI. The relative abundance of *V. dispar* was negatively associated with the concentration of LNnT and the relative abundance of *P. distasonis* was negatively associated with the concentrations of DFL and LNDFH-I. The result of the crude model is shown in Supplemental Figure S2.

**Figure 3:**
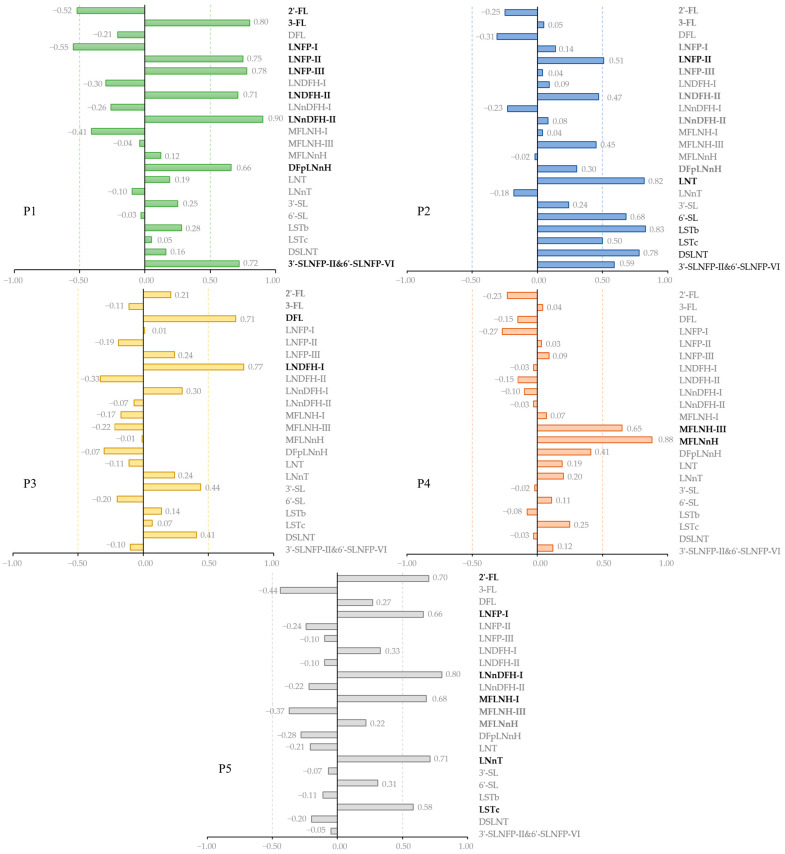
Factor loadings of different HMO patterns. Different colours indicate different patterns. HMOs with a factor loading of ≥0.5 or ≤−0.5 were taken as representative HMOs for each pattern and presented in bold font. P1–P5: HMOs Pattern 1–5. 2′-FL: 2′-fucosyllactose, 3-FL: 3-fucosyllactose, DFL: difucosyllactose, LNFP-I: lacto-*N*-fucopentaose I, LNFP-II: lacto-*N*-fucopentaose II, LNFP-III: lacto-*N*-fucopentaose III, LNDFH-I: lacto-*N*-difucohexaose I, LNDFH-II: lacto-*N*-difucohexaose II, LNnDFH-I: lacto-*N*-neodifucohexaose I, LNnDFH-II: lacto-*N*-neodifucohexaose II, MFLNH-I: monofucosyl-lacto-*N*-hexaose I, MFLNH-III: monofucosyl-lacto-*N*-hexaose III, MFLNnH: monofucosyl-lacto-*N*-neohexaose, DFpLNnH: difucosyl-para-lacto-*N*-neohexaose, LNT: lacto-*N*-tetraose, LNnT: lacto-*N*-neotetraose, 3′-SL: 3′-sialyllactose, 6′-SL: 6′-sialyllactose, LSTb: sialyllacto-*N*-tetraose b, LSTc: sialyllacto-*N*-tetraose c, DSLNT: disialyllacto-*N*-tetraose, 3′-SLNFP-II: 3-sialyl-latco-*N*-fucopentaose II, 6′-SLNFP-VI: 6-sialyl-latco-*N*-fucopentaose VI.

To explore how the HMO complex plays a role in mediating the infant gut microbiome, principal component analysis (PCA) was performed. With a varimax-rotation method, we drafted five different HMO patterns (Figure 3).

Pattern 1 (P1) was characterised by high levels of 3-FL, LNFP-II, LNFP-III, LNDFH-II, LNnDFH-II, DFpLNnH, and 3′-SLNFP-II&6′-SLNFP-VI as while as low levels of 2′-FL and LNFP-I. Pattern 2 (P2) was characterised by high levels of LNFP-II, LNT, 6′-SL, LSTb, LSTc, DSLNT, and 3′-SLNFP-II&6′-SLNFP-VI. Pattern 3 (P3) was characterised by high levels of DFL and LNDFH-I. Pattern 4 (P4) was characterised by high levels of MFLNH-III and MFLNnH. Pattern 5 (P5) was characterised by high levels of 2′-FL, LNFP-I, LNnDFH-I, MFLNH-I, LNnT, and LSTc. Altogether, these HMO patterns explained 78% of the total variances.

**Figure 4:**
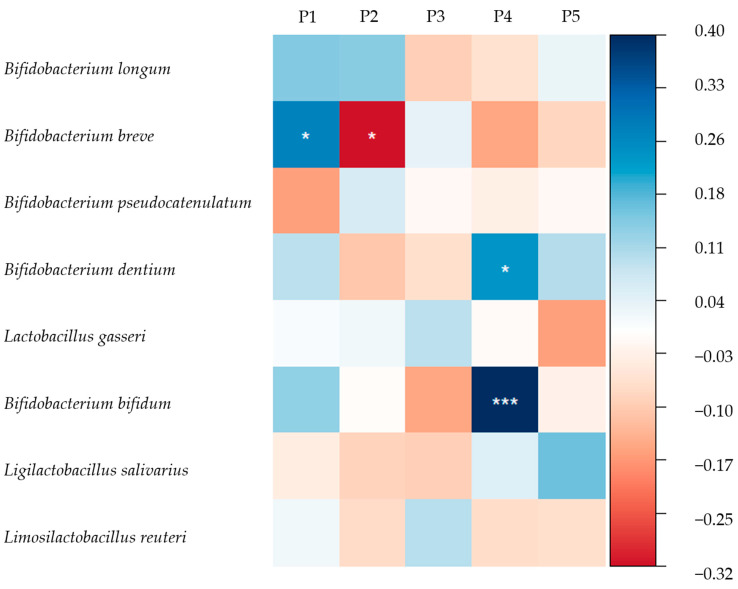
Standardised coefficients between HMO pattern scores and relative abundance of *Bifidobacterium* and *Lactobacilli*. *: *p* < 0.05, ***: *p* < 0.001. The eight most abundant infant gut microbiota from *Bifidobacterium* and *Lactobacilli* were explored. Adjusted for city, maternal age, total concentration of HMOs, and delivery mode. The relative abundance of infant gut microbiota was transformed in the form of lg (X + 1).

Subsequently, a general linear regression was conducted to explore the association between HMO pattern scores and the transformed relative abundance of infant gut microbiota. As shown in Figure 4, when city, maternal age, total concentration of HMOs, and delivery mode were controlled for, the relative abundance of *B. breve* was positively associated with the P1 score but negatively associated with the P2 score. 

**Figure 5:**
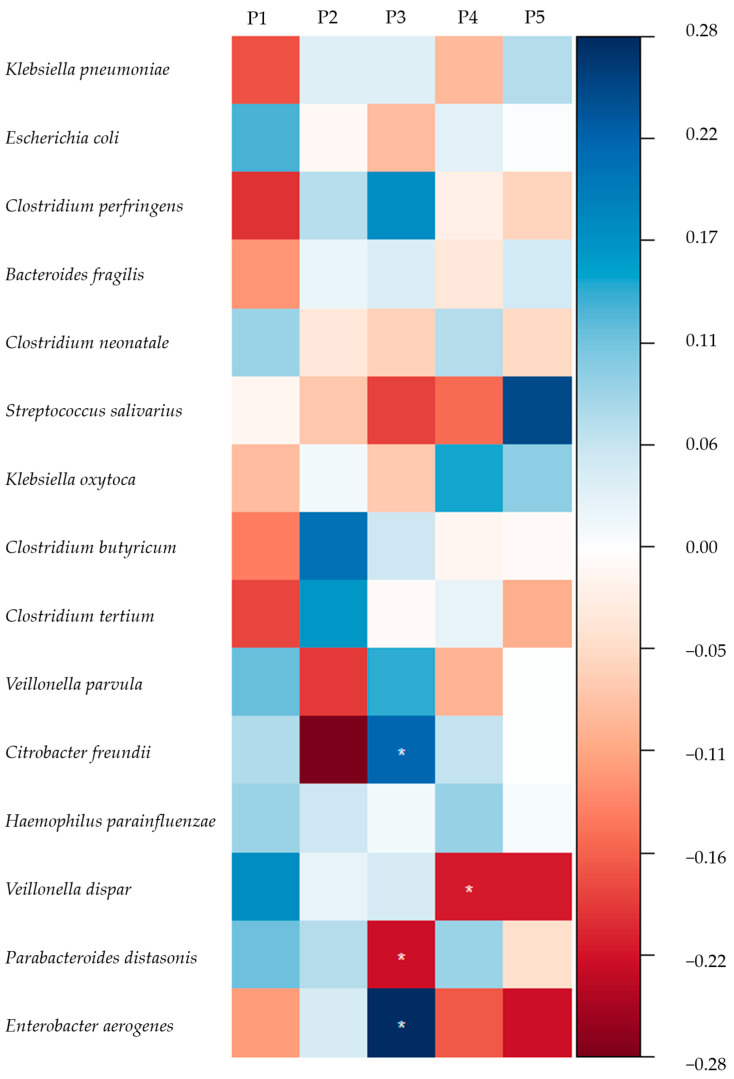
Standardised coefficients between HMO pattern scores and relative abundance of commensal bacteria. *: *p* < 0.05. The 15 most abundant infant gut microbiota except for *Bifidobacterium* and *Lactobacilli* were explored. Adjusted for city, maternal age, total concentration of HMOs, and delivery mode. The relative abundance of infant gut microbiota was transformed in the form of lg (X + 1).

The relative abundances of *B. dentium* and *B. bifidum* were positively associated with the P4 score. The results of the crude model are shown in Supplemental Figure S3. As for other commensal bacteria in the infant gut (Figure 5), after adjustment, the relative abundances of *C. freundii* and *E. aerogenes* were positively associated with the P3 score, while *P. distasonis* abundance was negatively related with P3. The relative abundance of *V. dispar* was negatively associated with the P4 score. The results of the crude model are shown in Supplemental Figure S4.

## 4. Discussion

In this study, we explored the association of different levels and patterns of HMOs in mature milk with the composition of infant gut microbiota using paired maternal–infant data. To the best of our knowledge, this study is the first in a Chinese population to report certain patterns of HMOs other than the patterns induced by maternal Lewis gene or Secretor gene, might also contribute to the variation of infant gut microbiome. In particular, the HMO pattern characterised by high levels of 3-FL, LNFP-II, LNFP-III, LNDFH-II, LNnDFH-II, DFpLNnH, and 3′-SLNFP-II&6′-SLNFP-VI, as well as low levels of 2′-FL and LNFP-I, was positively associated with *B. breve*. The HMO pattern characterised by high levels of MFLNH-III and MFLNnH was positively associated with *B. dentium* and *B. bifidum.*

### 4.1. HMOs Concentrations

Previous studies have shown that there are two main types of genes that determine the differences in HMO concentration: the Lewis gene and the Secretor gene [14,32,33]. Due to limitations in sample accessibility and sample size, only the secretor phenotype of maternal subjects was identified based on the presence of 2′-FL, DFL, and LNFP-II in this study, and 72.9% of the maternal subjects were Se. Our results are similar to those of studies conducted in Liaoning (76.8%) [15] and Guangdong (77.0%) [17], but lower than those of a study that included Beijing, Xuchang, and Suzhou (81.0%) [16]. The higher concentration of total HMOs and α-1,2 glycosidic fucosylated HMOs in Se mothers has been well established in previous studies [24]. We observed a non-significantly higher total concentration of HMOs in nSe mothers and all of the α-1,2 glycosidic fucosylated HMOs concentrations were extremely low or below the LOD in nSe samples, while other fucosylated HMOs, except for MFLNnH, were higher in nSe mothers. A higher concentration of LNT in nSe mothers and a higher concentration of LNnT in Se mothers were observed in this study. Previous studies have yielded various results on the concentrations of LNT and LNnT in Se/nSe mothers. Wu et al. [17] reported no differences between maternal secretor phenotypes in 59 Chinese mother–infant dyads, while a study including 427 Canadian mother–infant dyads reported higher concentrations of LNT and LNnT in nSe mothers [24]. Regarding sialylated HMOs, higher concentrations of LSTb, DSLNT, and 3′-SLNFP-II&6′-SLNFP-VI were detected in nSe mothers in this study. Meanwhile, a higher concentration of LSTc in Se mothers, along with no significant difference in 3′-SL, 6′-SL, LSTb, and DSLNT, was reported by Wu et al. [17]. In addition to the influencing factors mentioned earlier (such as maternal age, geographic location, and parity), these inconsistent conclusions may also stem from heterogeneity in the study population and the biases caused by sampling methods. 

In terms of nongenetic factors, the stage of milk maturation is one of the most important influencing factors. Previous studies have reported that the total concentration of HMOs in mature milk was approximately 5–20 g/L [34,35,36,37,38]. In our study, the mean of total concentration of HMOs was consistent with previous reports. For individual HMOs, 2′-FL was the most abundant HMOs in our study, which is also consistent with previous reports [20,39]. However, Zhang et al. [40] pointed out that 6′-SL was the most abundant HMO in Chinese mature milk from one-month postpartum. As mentioned, this difference might emerge from the hetero distribution of maternal and infant characteristics such as geographical location, maternal age, and parity between our study and theirs. Another momentous perspective is that, despite the similar procedures applied in the milk sample collection, the bias caused by the unidentical oligosaccharides standards used and the dissimilar approaches of HMO detection could impact the results [29,41]. It is interesting that a study using the high-performance anion-exchange chromatography-pulsed amperometric detector method detected 2′-FL in 99.8% of the Chinese milk samples [42]. Therefore, the detection method of HMOs, especially the impact of the detection limit of HMOs on the results, also calls for attention from researchers.

Few previous studies have been performed in China on patterns in HMOs other than the Se/nSe pattern. Using multiple factor analysis, Jiang et al. [43] drafted three mature milk factors that possessed apparent diversity compared to our results. In their study, 12 types of HMOs including 2′-FL, 3-FL, 3′-SL, 6′-SL, LNFP-I, LNFP-III, LNDFH-II, DSLNT, lacto-*N*-neohexaose (LNnH), LSTa, LSTb and LSTc were quantified and analysed. Factor 1 was positively associated with LNnH and LNDFH-II but negatively associated with 3′-SL. Factor 2 was positively associated with LNnH, sialyllacto-*N*-tetraose a (LSTa), LSTb, LSTc, 3-FL, and 2′-FL, but it was negatively associated with DSLNT. Factor 3 was positively associated with LSTc and LNFP-III and negatively associated with 2′-FL and 6′-SL. The distinct differences between their results and ours may be attributed to the different methods used to extract the patterns. Although both factor analysis and PCA are commonly applied to perform data dimension reduction, the result of the PCA method is a set of linearly uncorrelated variables generated though orthogonal transformation from the original data. And, in contrast with the factor analysis, such results could be artifacts of the method and may not actually reflect the biological phenomenon. In addition, the cities from which our samples and theirs (Chengdu, Guangzhou, Beijing, Weihai, Jinhua, and Lanzhou) were collected are not geographically consistent; the influence of geographic difference should be considered, as mentioned earlier [22]. Furthermore, the differences in quantity and methodology of HMO detection may also contribute to the discrepant results [29,41]. It is also of note that their study incorporated proteomics and lipidomics into the analysis, as well as HMOs, whereas our study presented patterns more concentrated on HMOs. The scarce and discordant nature of studies on HMO patterns in China demonstrates the need for more large-scaled studies with consistent analysis methods. Notably, Jiang et al. [43] did not delve into the impact of these HMO patterns on the infant gut microbiota.

### 4.2. Infant Gut Microbiota

Our result at phylum level is similar to that of Shang et al. [25]. However, in their study on vaginally delivered infants, the dominant microbiota at genus level were *Bifidobacterium*, *Clostridium*, *Streptococcus*, *Bacteroides*, *Klebsiella*, *Veillonella*, *Lactobacilli*, and *Akkermansia*. The differences might stem from the different delivery modes of the subjects. Previous studies had shown that for infants who underwent vaginal delivery, the gut was dominated by *Bacteroides*, *Bifidobacterium*, *Parabacteroides*, and *Escherichia*, while for infants who underwent cesarean section, *Klebsiella*, *Clostridia*, *Enterobacter*, *Staphylococcus*, and some opportunistic pathogens dominated [44]. Our study included both infants who underwent vaginal delivery and those who underwent cesarean section. Therefore, the dominant genera in our study included *Escherichia Shigella*, *Bacteroides*, and *Bifidobacterium* which were dominant in the vaginal-delivered infants, as well as *C. sensu stricto 1* and *Klebsiella* that were dominant in cesarean-section-delivered infants.

### 4.3. Association of HMO Patterns in Mature Milk with Infant Gut Microbiota

HMOs, in the form of individual HMOs or the pattern defined by the maternal phenotype, have long been investigated for their functions in immunomodulation, intestinal barrier enhancement, prebiotic effects, anti-adhesion, and infant neurodevelopment [45,46]. Previous studies have demonstrated that HMOs can provide abundant fucose and lactose, thereby affecting the infant gut microbiota through direct energy supply and cross feeding [10]. 

#### 4.3.1. Association of Se/nSe Pattern with Infant Gut Microbiota

The maternal secretor phenotype could determine the pattern of HMOs and subsequently influence the utilisation of HMOs by the infant gut microbiota. In this study, when cities, maternal age, delivery mode, and total concentration of HMOs were adjusted for, the infants who were nourished by Se mothers exhibited a lower relative abundance of *B. bifidum* compared to those nourished by nSe mothers. As the total HMO concentration was slightly higher in the nSe mothers than in the Se mothers, our results could be supported by those of Katoh et al. [47], who reported that *B. bifidum* possessed higher number of extracellular glycoside hydrolases for HMO degradation than other *Bifidobacterium* strains, and Hu et al. [48], who suggested that *B. bifidum* was able to metabolise most of the HMOs, while *B. longum* and *B. breve* mainly fed on LNT, LNnT (*B. breve* only), and lacto-*N*-biose I (LNB). It is of note that HMOs are not the only source of fucose in infants. In a recent study, researchers reported that the secretor phenotype of the infant subjects aged 6 to 9 weeks could affect the fucose content of their intestinal mucosa and thus regulate the composition of their gut microbiota. The study also suggested that the correlation between the gut microbiota of infants and their secretor phenotypes was stronger than that with their mothers’ secretor phenotypes [49]. A unanimous conclusion regarding the association between maternal secretor phenotype and infant gut microbiota is yet to be reached. The colonisation of *Bifidobacterium* in infants nourished by nSe mothers was shown to be delayed compared to those fed by Se mothers [50]. Wang et al. [46] suggested that the maternal secretor phenotype was not associated with the relative abundance of the gut microbiota, but infants fed by Se mothers exhibited a higher level of faecal 1,2-propanediol, which was produced by *Bifidobacterium* species in fermentation [51], compared to infants fed by nSe mothers. Meanwhile, Turpin et al. [27] suggested that for healthy infants, the secretor phenotype of their mothers was not associated with their gut microbiota composition, a finding supported by a study by Ramani et al. [28]. Hence, when it comes to application-oriented explorations, the association between HMO patterns other than a prior Se/nSe pattern and infant gut microbiota is an area of interest for future study.

#### 4.3.2. Association of Other HMO Patterns with Infant Gut Microbiota

Beyond the Se/nSe pattern, recent progress in HMO synthesis has provided researchers and enterprises with more possibilities for the combined use of HMOs [52]. We therefore performed PCA and drafted five HMO patterns which explained 78% of the total variances. After adjusting for cities, maternal age, delivery mode, and total HMO concentration, having a P1 pattern was positively associated with the relative abundance of *B. breve*, while the P2 pattern was negatively associated with *B. breve*. The *B. breve* count in colostrum samples was positively correlated to both LNFP-III and LSTc in a previous study [53]. Additionally, LNT was one of the representative HMOs in P2 and is known to be one of the major HMOs catabolised by *B. breve* in vitro [54]. However, a negative association between P2 and *B. breve* was observed in this study. The inconsistencies may indicate that other factors beyond HMOs such as host-secreted glycans [55] or colonisation order of the *Bifidobacterium* species [56] have a greater influence on the infant gut microbiota. Furthermore, other commensal microbiota such as *Bacteroides* and *Lactobacilli* also possess the ability to degrade HMOs [57] and the competitive colonisation between these genera may also have an impact on our results. The P4 pattern was positively associated with *B. dentium* and *B. bifidum*. These associations were consistent with the individual-level exploration in this study. The ability of *B. dentium* isolated from infant faeces to produce β-galactosidases hydrolysing LNT and LNnT has been demonstrated in vitro [58], and the structural similarities of MFLNH-III with LNT and MFLNnH with LNnT might contribute to the explanation of our results. The broad HMO metabolic spectrum of *B. bifidum* was mentioned previously, but the mechanism of its unique association with MFLNH-III, MFLNnH, and DFpLNnH in this study is yet to be examined. Moreover, the pathways deployed by *Bifidobacterium* to degrade HMOs differed at the species and even strain level [59,60]. Given that the sequencing method used in this study was unable to reach the strain level, the impact of strain-level differences on the results also requires consideration. And again, it is vital to underline the statistically generated nature of the results of PCA, which may lead to misinterpretations and inappropriate presentation of the associations of the HMO patterns we drafted with infant gut microbiota.

At present, HMOs such as 2′-FL and LNnT have been approved for use in infant formula in countries and regions across the world [61]. Given that HMOs ingested by infants through breast milk are, in fact, a complex of structurally similar and diverse oligosaccharides rather than individual HMOs, exploring the association between specific HMO patterns and infant gut microbiota could provide additional insights for subsequent research and commercial applications. Taken together, our results suggest that HMO patterns in mature milk, other than the Se/nSe pattern, are associated with certain gut microbiota, especially *Bifidobacterium*, in infants.

### 4.4. Strengths and Limitations

A strength of our study is that all infant subjects were exclusively breastfed by their mothers. A further strength is that our milk samples were collected at least one-month post-partum, so there is no confounding caused by the differences in HMOs between colostrum and mature milk. Moreover, we detected 23 types of HMOs and explored how the HMO complex, in the form of the prior Se/nSe pattern and the five posteriori patterns, plays a role in mediating the infant gut microbiome, providing future studies and applications of combined usage of HMOs with a potential direction. 

There are several limitations to this study. Firstly, as a cross-sectional study, we could only estimate a recent association between HMOs and the infant gut microbiota. The time window in which HMOs affect the infant gut microbiota should be further determined. Secondly, there are residual confounders that may affect the results; other deep-seated drivers of HMOs patterns, therefore, require more attention. Thirdly, the 16SRNA method could at best determine the species level; the impact of strain-level differences on the results therefore also needs to be considered. Prospective studies with larger sample sizes and more precise sequencing methods such as metagenome are required to confirm the results of this study.

## 5. Conclusions

In conclusion, the association of different levels and patterns of HMOs in mature milk with the composition of the infant gut microbiota was explored in this study. Our results suggest that certain HMO patterns contribute to variation in the infant gut microbiome. Particularly, infants who were nourished by Se mothers exhibited a lower relative abundance of *B. bifidum*. The HMOs pattern characterised by high levels of 3-FL, LNFP-II, LNFP-III, LNDFH-II, LNnDFH-II, DFpLNnH, and 3′-SLNFP-II&6′-SLNFP-VI as well as low levels of 2′-FL and LNFP-I was positively associated with the relative abundance of *B. breve*. Furthermore, the HMO pattern characterised by high levels of MFLNH-III and MFLNnH was positively associated with the relative abundance of *B. dentium* and *B. bifidum*. Such results provide future studies and applications of combined usage of HMOs with a potential direction.

## Figures and Tables

**Table 1:** Distribution of HMOs across maternal secretor phenotypes (mg/L).

HMOs	Se (*n* = 70)	nSe (*n* = 26)	*p*
2′-FL	1760.5 (1405.0, 2188.3)	-	-
3-FL ^a^	454.0 (317.3, 638.5)	1522.7 (1181.0, 1871.8)	<0.001
DFL	216.4 (144.5, 271.7)	-	-
LNFP-I	447.3 (252.1, 804.5)	-	-
LNFP-II ^a^	154.8 (94.0, 250.3)	992.7 (834.9, 1191.8)	<0.001
LNFP-III ^a^	281.0 (226.2, 335.2)	403.5 (320.1, 464.8)	<0.001
LNDFH-I ^a^	479.9 (318.5, 639.9)	3.9 (0.0, 21.1)	<0.001
LNDFH-II ^a^	0.0 (0.0, 35.2)	179.7 (119.1, 328.2)	<0.001
LNnDFH-I	28.2 (19.1, 37.1)	-	-
LNnDFH-II ^a^	22.4 (15.7, 34.0)	90.8 (59.1, 116.7)	<0.001
MFLNH-I	48.0 (17.9, 98.4)	-	-
MFLNH-III ^a^	178.0 (120.6, 243.0)	423.7 (225.4, 626.9)	<0.001
MFLNnH ^a^	97.8 (57.1, 154.4)	96.4 (50.1, 215.7)	0.677
DFpLNnH ^a^	116.4 (84.1, 140.3)	361.2 (278.9, 465.4)	<0.001
LNT ^a^	374.9 (279.7, 578.8)	896.2 (652.5, 1081.7)	<0.001
LNnT ^a^	104.9 (65.0, 136.3)	36.4 (18.2, 51.7)	<0.001
3′-SL ^a^	88.7 (81.1, 106.1)	101.8 (86.5, 112.9)	0.124
6′-SL ^a^	249.6 (158.4, 348.9)	284.2 (226.7, 391.9)	0.213
LSTb ^a^	41.4 (26.3, 57.9)	77.1 (62.0, 102.6)	<0.001
LSTc ^b^	133.9 ± 63.6	125.4 ± 50.9	0.503
DSLNT ^a^	172.8 (121.0, 257.6)	281.4 (217.7, 416.4)	<0.001
3′-SLNFP-II&6′-SLNFP-VI ^a^	33.8 (24.3, 54.3)	91.3 (75.9, 119.4)	<0.001
Sum ^b^	6063.2 ± 1299.9	6232.8 ± 1376.3	0.589

^a^: presented as median (P25, P75) and tested with Kruskall–Wallis test, ^b^: presented as mean ± SD and tested with *t*-test, HMOs: human milk oligosaccharides, 2′-FL: 2′-fucosyllactose, 3-FL: 3-fucosyllactose, DFL: difucosyllactose, LNFP-I: lacto-*N*-fucopentaose I, LNFP-II: lacto-*N*-fucopentaose II, LNFP-III: lacto-*N*-fucopentaose III, LNDFH-I: lacto-*N*-difucohexaose I, LNDFH-II: lacto-*N*-difucohexaose II, LNnDFH-I: lacto-*N*-neodifucohexaose I, LNnDFH-II: lacto-*N*-neodifucohexaose II, MFLNH-I: monofu-cosyl-lacto-*N*-hexaose I, MFLNH-III: monofucosyl-lacto-*N*-hexaose III, MFLNnH: monofucosyl-lacto-*N*-neohexaose, DFpLNnH: difucosyl-para-lacto-*N*-neohexaose, LNT: lac-to-*N*-tetraose, LNnT: lacto-*N*-neotetraose, 3′-SL: 3′-sialyllactose, 6′-SL: 6′-sialyllactose, LSTb: sialyllacto-*N*-tetraose b, LSTc: sialyllacto-*N*-tetraose c, DSLNT: disialyllac-to-*N*-tetraose, 3′-SLNFP-II: 3-sialyl-latco-*N*-fucopentaose II, 6′-SLNFP-VI: 6-sialyl-latco-*N*-fucopentaose VI, Sum: total concentration of the HMOs detected.

**Table 2:** Associations between maternal secretor phenotype and *Bifidobacterium* and *Lactobacilli* β (95%CI) ^a^.

Species	Crude Model	Model 1
*Bifidobacterium longum*	−0.088 (−0.292, 0.116)	−0.161 (−0.385, 0.063)
*Bifidobacterium breve*	−0.129 (−0.332, 0.074)	−0.111 (−0.334, 0.113)
*Bifidobacterium pseudocatenulatum*	0.132 (−0.071, 0.335)	0.165 (−0.058, 0.388)
*Bifidobacterium dentium*	−0.056 (−0.261, 0.148)	−0.128 (−0.353, 0.097)
*Lactobacillus gasseri*	0.039 (−0.166, 0.244)	0.013 (−0.207, 0.233)
*Bifidobacterium bifidum*	−0.160 (−0.362, 0.042)	−0.245 (−0.465, −0.025)
*Ligilactobacillus salivarius*	0.065 (−0.140, 0.269)	0.032 (−0.196, 0.259)
*Limosilactobacillus reuteri*	0.087 (−0.117, 0.291)	0.063 (−0.154, 0.281)

The eight most abundant infant gut microbiota from *Bifidobacterium* and *Lactobacilli* were explored. Crude model: only the maternal secretor phenotype was included. Model 1: Adjusted for city (Chengdu, Guangzhou, Hohhot, Beijing, Suzhou), maternal age (≤30 y or >30 y), total concentration of HMOs (mg/L), and mode of delivery (vaginal delivery or caesarean section). ^a^: results of linear regression with nSe mothers as reference, the relative abundance of infant gut microbiota was transformed in the form of lg (X + 1).

## Data Availability

The data presented in this study are available on request from the corresponding author. The data are not publicly available due to ethical requirements.

## Supplementary Materials

The following supporting information can be downloaded at: https://www.mdpi.com/article/10.3390/nu16091287/s1, Table S1. Association between maternal secretor phenotype and commensal bacteria; Figure S1. Standardised coefficients between individual HMO concentrations and relative abundance of *Bifidobacterium* and *Lactobacilli* in crude model; Figure S2. Standardised coefficients between individual HMO concentrations and relative abundance of commensal bacteria; Figure S3. Standardised coefficients between HMO pattern scores and relative abundance of *Bifidobacterium* and *Lactobacilli* in crude model, Figure S4. Standardised coefficients between HMO pattern scores and relative abundance of commensal bacteria.

## Appendix A

**Table A1:** Distribution of HMOs across maternal age groups (mg/L).

HMOs	≤30 (*n* = 42)	>30 (*n* = 54)	*p*
2′-FL ^a^	954.5 (0.0, 1730.6)	1685.8 (1278.1, 2097.3)	0.011 ^a^
3-FL ^a^	911.2 (358.4, 1475.9)	514.3 (339.9, 740.8)	0.063 ^a^
DFL ^a^	95.4 (0.0, 214.2)	211.6 (125.0, 260.2)	0.002 ^a^
LNFP-I ^a^	75.2 (0.0, 547.0)	401.7 (168.8, 731.9)	0.009 ^a^
LNFP-II ^a^	290.8 (103.0, 883.9)	168.4 (107.1, 345.0)	0.234 ^a^
LNFP-III ^a^	329.2 (258.5, 410.5)	289.8 (229.1, 339.9)	0.049 ^a^
LNDFH-I ^a^	47.3 (0.0, 526.9)	428.6 (298.0, 608.4)	0.011 ^a^
LNDFH-II ^a^	40.6 (0.0, 138.6)	9.2 (0.0, 54.7)	0.136 ^a^
LNnDFH-I ^a^	14.1 (0.0, 29.3)	27.1 (17.0, 36.9)	0.006 ^a^
LNnDFH-II ^a^	41.4 (21.1, 82.7)	23.6 (16.8, 36.4)	0.032 ^a^
MFLNH-I ^a^	1.5 (0.0, 59.8)	35.5 (10.9, 84.5)	0.024 ^a^
MFLNH-III ^a^	241.5 (153.1, 388.9)	193.2 (120.1, 258.1)	0.083 ^a^
MFLNnH ^a^	97.7 (59.0, 183.0)	96.9 (55.6, 152.9)	0.679 ^a^
DFpLNnH ^a^	171.2 (113.6, 282.6)	124.3 (92.7, 183.0)	0.045 ^a^
LNT ^a^	556.1 (300.1, 899.3)	456.8 (294.3, 687.8)	0.305 ^a^
LNnT ^a^	53.6 (32.1, 90.2)	96.6 (54.6, 136.3)	0.016 ^a^
3′-SL ^a^	91.4 (81.8, 106.1)	91.3 (83.5, 108.9)	0.621 ^a^
6′-SL ^a^	250.6 (185.5, 315.6)	259.1 (156.0, 410.0)	0.296 ^a^
LSTb ^a^	53.7 (29.6, 77.2)	50.7 (30.7, 73.5)	0.805 ^a^
LSTc ^b^	131.1 ± 64.6	132.0 ± 57.2	0.945 ^b^
DSLNT ^a^	208.5 (150.3, 306.4)	184.1 (133.0, 286.7)	0.545 ^a^
3′-SLNFP-II&6′-SLNFP-VI ^a^	60.1 (29.2, 83.7)	35.3 (25.3, 60.9)	0.080 ^a^
Sum ^b^	6025.8 ± 1417.0	6174.0 ± 1241.2	0.593 ^b^

^a^: presented as median (P25, P75) and tested with Kruskall–Wallis test, ^b^: presented as mean ± SD and tested with *t*-test, HMOs: human milk oligosaccharides, 2′-FL: 2′-fucosyllactose, 3-FL: 3-fucosyllactose, DFL: difucosyllactose, LNFP-I: lacto-*N*-fucopentaose I, LNFP-II: lacto-*N*-fucopentaose II, LNFP-III: lacto-*N*-fucopentaose III, LNDFH-I: lacto-*N*-difucohexaose I, LNDFH-II: lacto-*N*-difucohexaose II, LNnDFH-I: lacto-*N*-neodifucohexaose I, LNnDFH-II: lacto-*N*-neodifucohexaose II, MFLNH-I: monofu-cosyl-lacto-*N*-hexaose I, MFLNH-III: monofucosyl-lacto-*N*-hexaose III, MFLNnH: monofucosyl-lacto-*N*-neohexaose, DFpLNnH: difucosyl-para-lacto-*N*-neohexaose, LNT: lac-to-*N*-tetraose, LNnT: lacto-*N*-neotetraose, 3′-SL: 3′-sialyllactose, 6′-SL: 6′-sialyllactose, LSTb: sialyllacto-*N*-tetraose b, LSTc: sialyllacto-*N*-tetraose c, DSLNT: disialyllac-to-*N*-tetraose, 3′-SLNFP-II: 3-sialyl-latco-*N*-fucopentaose II, 6′-SLNFP-VI: 6-sialyl-latco-*N*-fucopentaose VI, Sum: total concentration of the HMOs detected.

**Table A2:** Distribution of HMOs across cities (mg/L).

HMOs	Chengdu (*n* = 15)	Guangzhou (*n* = 10)	Hohhot (*n* = 14)	Suzhou (*n* = 18)	Beijing (*n* = 39)	*p*
2′-FL ^a^	0.0 (0.0, 1687.1)	1378.7 (1005.0, 2336.0)	1445.2 (298.4, 1588.5)	1529.6 (800.5, 1758.4)	1882.3 (940.4, 2170.3)	0.224 ^a^
3-FL ^a^	612.9 (376.1, 1416.1)	694.5 (390.0, 885.3)	867.4 (587.8, 1521.9)	550.2 (338.8, 1072.2)	454.7 (302.9, 894.3)	0.066 ^a^
DFL ^a^	0.0 (0.0, 178.7)	202.2 (51.2, 242.5)	217.6 (47.0, 296.9)	201.3 (104.8, 254.8)	143.5 (64.2, 244.0)	0.224 ^a^
LNFP-I ^a^	0.0 (0.0, 441.8)	196.9 (62.5, 781.8)	153.7 (7.2, 280.6)	320.3 (87.7, 688.5)	558.2 (179.1, 803.9)	0.025 ^a^
LNFP-II ^a^	418.9 (112.4, 936.9)	144.1 (105.9, 490.0)	268.9 (163.3, 476.3)	165.7 (98.8, 256.6)	230.2 (98.9, 405.0)	0.516 ^a^
LNFP-III ^a^	371.0 (330.0, 407.0)	299.4 (279.3, 330.8)	319.6 (281.0, 390.1)	241.6 (193.5, 313.5)	281.2 (231.2, 374.5)	0.010 ^a^
LNDFH-I ^a^	39.1 (21.5, 482.3)	335.4 (69.5, 623.0)	458.7 (116.1, 599.5)	312.0 (55.0, 433.4)	411.8 (0.0, 636.7)	0.795 ^a^
LNDFH-II ^a^	10.5 (0.0, 172.9)	0.0 (0.0, 43.1)	23.2 (3.5, 103.0)	26.9 (7.8, 50.7)	0.0 (0.0, 114.2)	0.715 ^a^
LNnDFH-I ^a^	0.0 (0.0, 26.3)	17.5 (8.8, 21.1)	19.2 (3.9, 25.8)	18.9 (4.6, 27.5)	32.9 (14.9, 42.4)	0.065 ^a^
LNnDFH-II ^a^	38.7 (20.2, 78.2)	25.7 (17.0, 35.0)	41.4 (25.3, 82.4)	24.8 (11.0, 43.7)	33.5 (17.0, 45.4)	0.175 ^a^
MFLNH-I ^a^	0.0 (0.0, 30.1)	13.0 (2.7, 36.7)	21.1 (0.0, 53.2)	13.9 (0.0, 86.5)	56.5 (5.4, 119.2)	0.109 ^a^
MFLNH-III ^a^	195.3 (161.4, 400.4)	224.9 (147.1, 254.1)	237.3 (172.5, 307.6)	133.1 (96.4, 174.3)	222.3 (174.1, 282.2)	0.079 ^a^
MFLNnH ^a^	97.6 (74.2, 129.2)	94.2 (82.8, 119.9)	154.7 (97.1, 217.8)	70.8 (53.5, 109.5)	119.3 (47.2, 179.6)	0.321 ^a^
DFpLNnH ^a^	152.3 (119.0, 284.5)	121.0 (84.8, 135.4)	183.4 (134.1, 220.9)	98.9 (84.4, 159.7)	139.9 (98.2, 219.5)	0.142 ^a^
LNT ^a^	605.9 (430.6, 839.5)	307.6 (271.2, 393.0)	399.4 (266.2, 553.5)	420.8 (296.2, 613.8)	650.0 (324.6, 933.8)	0.059 ^a^
LNnT ^a^	52.7 (22.5, 113.5)	54.5 (30.5, 82.4)	85.0 (30.3, 102.9)	82.0 (50.9, 129.3)	86.0 (48.0, 136.1)	0.479 ^a^
3′-SL ^a^	102.2 (85.8, 119.2)	90.9 (83.3, 104.8)	84.9 (73.1, 95.3)	87.4 (82.7, 100.4)	94.9 (82.3, 112.1)	0.535 ^a^
6′-SL ^a^	225.5 (153.8, 279.5)	240.3 (113.7, 254.4)	206.9 (133.1, 290.4)	199.4 (144.1, 288.8)	361.6 (259.1, 447.1)	<0.001 ^a^
LSTb ^a^	66.6 (42.5, 82.9)	35.1 (27.9, 48.9)	47.2 (17.3, 54.5)	40.0 (24.8, 54.6)	64.8 (40.3, 83.2)	0.009 ^a^
LSTc ^b^	147.6 ± 59.9	110.5 ± 56.0	122.7 ± 70.7	105.0 ± 60.0	146.3 ± 53.7	0.074 ^b^
DSLNT ^a^	235.3 (201.0, 431.9)	197.0 (140.8, 288.9)	186.2 (123.9, 281.7)	126.4 (79.9, 178.4)	203.2 (166.5, 325.6)	0.002 ^a^
3′-SLNFP-II&6′-SLNFP-VI ^a^	59.4 (32.4, 83.1)	31.2 (22.6, 59.4)	45.8 (27.1, 60.8)	32.2 (23.7, 39.8)	54.4 (33.1, 82.0)	0.065 ^a^
Sum ^b^	5833.8 ± 1050.7	6003.8 ± 1692.3	5893.3 ± 1161.2	5271.4 ± 1122.4	6706.2 ± 1212.8	0.002 ^b^

^a^: presented as median (P25, P75) and tested with Kruskall–Wallis test, ^b^: presented as mean ± SD and tested with *t*-test, HMOs: human milk oligosaccharides, 2′-FL: 2′-fucosyllactose, 3-FL: 3-fucosyllactose, DFL: difucosyllactose, LNFP-I: lacto-*N*-fucopentaose I, LNFP-II: lacto-*N*-fucopentaose II, LNFP-III: lacto-*N*-fucopentaose III, LNDFH-I: lacto-*N*-difucohexaose I, LNDFH-II: lacto-*N*-difucohexaose II, LNnDFH-I: lacto-*N*-neodifucohexaose I, LNnDFH-II: lacto-*N*-neodifucohexaose II, MFLNH-I: monofu-cosyl-lacto-*N*-hexaose I, MFLNH-III: monofucosyl-lacto-*N*-hexaose III, MFLNnH: monofucosyl-lacto-*N*-neohexaose, DFpLNnH: difucosyl-para-lacto-*N*-neohexaose, LNT: lac-to-*N*-tetraose, LNnT: lacto-*N*-neotetraose, 3′-SL: 3′-sialyllactose, 6′-SL: 6′-sialyllactose, LSTb: sialyllacto-*N*-tetraose b, LSTc: sialyllacto-*N*-tetraose c, DSLNT: disialyllac-to-*N*-tetraose, 3′-SLNFP-II: 3-sialyl-latco-*N*-fucopentaose II, 6′-SLNFP-VI: 6-sialyl-latco-*N*-fucopentaose VI, Sum: total concentration of the HMOs detected.

**Table A3:** Distribution of HMOs across delivery modes (mg/L).

HMOs	Caesarean (*n* = 29)	Vaginal (*n* = 67)	*p*
2′-FL ^a^	1743.1 (1221.7, 2188.1)	1485.4 (0.0, 2037.2)	0.212 ^a^
3-FL ^a^	533.7 (324.9, 922.9)	627.5 (363.8, 1133.1)	0.478 ^a^
DFL ^a^	210.8 (102.8, 253.6)	143.5 (0.0, 246.7)	0.348 ^a^
LNFP-I ^a^	364.7 (135.2, 726.3)	249.8 (0.0, 661.1)	0.496 ^a^
LNFP-II ^a^	175.3 (104.4, 445.9)	229.5 (108.3, 728.8)	0.607 ^a^
LNFP-III ^a^	280.8 (243.5, 350.8)	311.6 (235.0, 381.9)	0.555 ^a^
LNDFH-I ^a^	473.4 (303.8, 593.5)	314.6 (7.6, 536.5)	0.064 ^a^
LNDFH-II ^a^	6.7 (0.0, 55.6)	29.4 (0.0, 123.7)	0.165 ^a^
LNnDFH-I ^a^	27.2 (17.6, 34.9)	19.1 (0.0, 35.2)	0.310 ^a^
LNnDFH-II ^a^	30.4 (18.9, 52.6)	33.5 (16.8, 53.6)	0.823 ^a^
MFLNH-I ^a^	37.4 (0.0, 105.3)	16.4 (0.0, 64.4)	0.084 ^a^
MFLNH-III ^a^	191.2 (168.1, 304.9)	196.4 (127.9, 301.6)	0.681 ^a^
MFLNnH ^a^	145.4 (95.2, 182.7)	89.5 (50.4, 148.1)	0.013 ^a^
DFpLNnH ^a^	133.2 (99.2, 205.9)	139.0 (95.3, 225.0)	0.883 ^a^
LNT ^a^	498.1 (307.3, 644.8)	456.3 (293.9, 859.1)	0.563 ^a^
LNnT ^a^	89.5 (59.8, 136.6)	66.4 (36.8, 120.5)	0.206 ^a^
3′-SL ^a^	94.9 (87.5, 119.7)	88.2 (80.6, 106.5)	0.070 ^a^
6′-SL ^a^	312.7 (196.7, 436.9)	248.4 (155.5, 347.9)	0.065 ^a^
LSTb ^a^	51.1 (29.5, 67.0)	51.8 (30.5, 77.1)	0.753 ^a^
LSTc ^b^	157.1 ± 65.6	120.6 ± 54.7	0.012 ^b^
DSLNT ^a^	189.5 (157.0, 273.1)	197.5 (129.3, 312.7)	0.820 ^a^
3′-SLNFP-II&6′-SLNFP-VI ^a^	40.9 (33.1, 64.3)	39.2 (25.1, 74.6)	0.555 ^a^
Sum ^b^	6258.1 ± 1150.8	6044.7 ± 1384.4	0.437 ^b^

^a^: presented as median (P25, P75) and tested with Kruskall–Wallis test, ^b^: presented as mean ± SD and tested with *t*-test, HMOs: human milk oligosaccharides, 2′-FL: 2′-fucosyllactose, 3-FL: 3-fucosyllactose, DFL: difucosyllactose, LNFP-I: lacto-*N*-fucopentaose I, LNFP-II: lacto-*N*-fucopentaose II, LNFP-III: lacto-*N*-fucopentaose III, LNDFH-I: lacto-*N*-difucohexaose I, LNDFH-II: lacto-*N*-difucohexaose II, LNnDFH-I: lacto-*N*-neodifucohexaose I, LNnDFH-II: lacto-*N*-neodifucohexaose II, MFLNH-I: monofu-cosyl-lacto-*N*-hexaose I, MFLNH-III: monofucosyl-lacto-*N*-hexaose III, MFLNnH: monofucosyl-lacto-*N*-neohexaose, DFpLNnH: difucosyl-para-lacto-*N*-neohexaose, LNT: lac-to-*N*-tetraose, LNnT: lacto-*N*-neotetraose, 3′-SL: 3′-sialyllactose, 6′-SL: 6′-sialyllactose, LSTb: sialyllacto-*N*-tetraose b, LSTc: sialyllacto-*N*-tetraose c, DSLNT: disialyllac-to-*N*-tetraose, 3′-SLNFP-II: 3-sialyl-latco-*N*-fucopentaose II, 6′-SLNFP-VI: 6-sialyl-latco-*N*-fucopentaose VI, Sum: total concentration of the HMOs detected.

**Table A4:** Distribution of HMOs across parity groups (mg/L).

HMOs	Primipara (*n* = 71)	Multipara (*n* = 25)	*p*
2′-FL ^a^	1568.0 (0.0, 2127.4)	1336.0 (1139.2, 1700.1)	0.495
3-FL ^a^	588.8 (360.4, 1168.3)	518.8 (317.9, 754.4)	0.341
DFL ^a^	177.1 (0.0, 257.0)	167.6 (102.8, 240.5)	0.960
LNFP-I ^a^	271.8 (0.0, 662.9)	404.5 (154.6, 705.8)	0.441
LNFP-II ^a^	229.5 (98.8, 728.8)	167.9 (134.3, 418.9)	0.809
LNFP-III ^a^	303.8 (238.2, 374.5)	300.4 (232.4, 378.9)	0.764
LNDFH-I ^a^	338.0 (11.9, 550.5)	394.6 (288.5, 556.7)	0.297
LNDFH-II ^a^	22.0 (0.0, 123.7)	11.4 (0.0, 55.6)	0.724
LNnDFH-I ^a^	20.7 (0.0, 35.1)	20.2 (6.8, 34.9)	0.983
LNnDFH-II ^a^	33.7 (18.4, 56.7)	28.8 (15.7, 36.7)	0.236
MFLNH-I ^a^	25.6 (0.0, 72.9)	24.5 (2.2, 84.8)	0.426
MFLNH-III ^a^	196.4 (132.9, 298.0)	191.2 (157.9, 313.7)	0.726
MFLNnH ^a^	96.5 (54.9, 173.3)	111.1 (59.9, 155.9)	0.943
DFpLNnH ^a^	139.9 (99.5, 225.6)	129.6 (86.9, 142.9)	0.299
LNT ^a^	445.3 (280.1, 700.7)	675.5 (413.3, 804.4)	0.130
LNnT ^a^	69.7 (39.2, 113.6)	107.1 (47.5, 142.6)	0.151
3′-SL ^a^	94.3 (82.7, 112.1)	85.9 (79.6, 101.2)	0.092
6′-SL ^a^	266.6 (175.6, 377.6)	248.8 (155.5, 369.4)	0.475
LSTb ^a^	51.1 (28.2, 71.0)	54.9 (39.1, 83.2)	0.172
LSTc ^b^	131.9 ± 58.1	130.6 ± 67.4	0.932
DSLNT ^a^	197.5 (145.9, 306.0)	184.1 (132.3, 266.6)	0.673
3′-SLNFP-II&6′-SLNFP-VI ^a^	40.9 (28.5, 77.0)	36.4 (23.9, 61.4)	0.455
Sum ^b^	6163.2 ± 1267.9	5955.8 ± 1460.3	0.532

^a^: presented as median (P25, P75) and tested with Kruskall–Wallis test, ^b^: presented as mean ± SD and tested with *t*-test, HMOs: human milk oligosaccharides, 2′-FL: 2′-fucosyllactose, 3-FL: 3-fucosyllactose, DFL: difucosyllactose, LNFP-I: lacto-*N*-fucopentaose I, LNFP-II: lacto-*N*-fucopentaose II, LNFP-III: lacto-*N*-fucopentaose III, LNDFH-I: lacto-*N*-difucohexaose I, LNDFH-II: lacto-*N*-difucohexaose II, LNnDFH-I: lacto-*N*-neodifucohexaose I, LNnDFH-II: lacto-*N*-neodifucohexaose II, MFLNH-I: monofu-cosyl-lacto-*N*-hexaose I, MFLNH-III: monofucosyl-lacto-*N*-hexaose III, MFLNnH: monofucosyl-lacto-*N*-neohexaose, DFpLNnH: difucosyl-para-lacto-*N*-neohexaose, LNT: lac-to-*N*-tetraose, LNnT: lacto-*N*-neotetraose, 3′-SL: 3′-sialyllactose, 6′-SL: 6′-sialyllactose, LSTb: sialyllacto-*N*-tetraose b, LSTc: sialyllacto-*N*-tetraose c, DSLNT: disialyllac-to-*N*-tetraose, 3′-SLNFP-II: 3-sialyl-latco-*N*-fucopentaose II, 6′-SLNFP-VI: 6-sialyl-latco-*N*-fucopentaose VI, Sum: total concentration of the HMOs detected.
