# Supplementary material for: Patterns of Human Milk Oligosaccharides in Mature Milk Are Associated with Certain Gut Microbiota in Infants
Source: Nutrients. 2024 Apr 25;16(9):1287. doi: 10.3390/nu16091287 (PMC11085179; doi:10.3390/nu16091287)
Supplement: Supplementary file 1 [file nutrients-16-01287-s001.zip › nutrients-2947636-supplementary.pdf]

Supplemental Table S1. Association between maternal secretor phenotype and commensal bacteria β(95%CI) [a]

Species	Crude model	Model 1
Klebsiella pneumoniae	0.015 (-0.190, 0.220)	0.101 (-0.094, 0.297)
Escherichia coli	-0.068 (-0.273, 0.136)	-0.111 (-0.328, 0.107)
Clostridium perfringens	0.086 (-0.118, 0.290)	0.137 (-0.073, 0.347)
Bacteroides fragilis	0.154 (-0.049, 0.356)	0.158 (-0.046, 0.363)
Clostridium neonatale	-0.161 (-0.363, 0.041)	-0.059 (-0.246, 0.129)
Streptococcus salivarius	0.034 (-0.171, 0.238)	0.085 (-0.136, 0.306)
Klebsiella oxytoca	0.041 (-0.163, 0.246)	-0.017 (-0.233, 0.200)
Clostridium butyricum	-0.028 (-0.233, 0.177)	0.020 (-0.207, 0.248)
Clostridium tertium	-0.050 (-0.255, 0.154)	-0.035 (-0.252, 0.182)
Veillonella parvula	0.004 (-0.201, 0.209)	-0.004 (-0.231, 0.223)
Citrobacter freundii	0.045 (-0.159, 0.250)	0.059 (-0.165, 0.282)
Haemophilus parainfluenzae	-0.007 (-0.212, 0.198)	0.020 (-0.206, 0.247)
Veillonella dispar	0.013 (-0.192, 0.217)	-0.07 (-0.293, 0.154)
Parabacteroides distasonis	-0.117 (-0.321, 0.086)	-0.222 (-0.431, -0.012)
Enterobacter aerogenes	0.126 (-0.077, 0.329)	0.098 (-0.128, 0.324)

Top 15 most abundant infant gut microbiota except for *Bifidobacterium* and *Lactobacilli* were explored. Crude model: only maternal secretor phenotype was included. Model 1: Adjusted for city (Chengdu, Guangzhou, Hohhot, Beijing, Suzhou), maternal age (≤ 30 y or > 30y), total concentration of HMOs (mg/L), and deliver mode (vaginal delivery or caesarean section). a: results of linear regression with nSe mothers as reference, the relative abundance of infant gut microbiota was transformed in the form of lg (X+1).

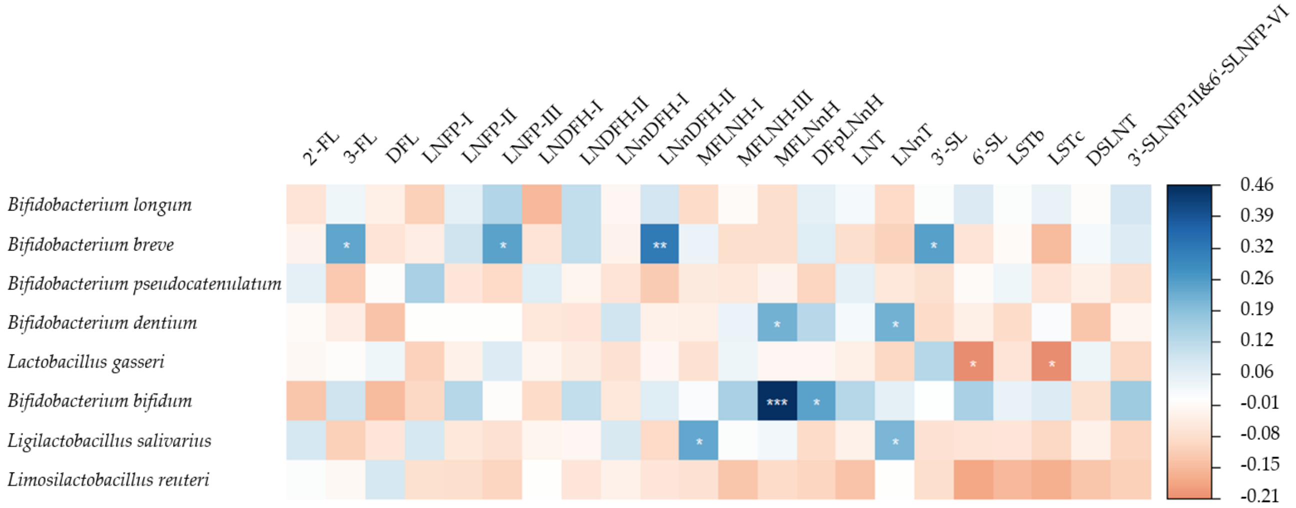

Supplemental Figure S1. Standardised coefficients between individual HMOs concentrations and relative abundance of *Bifidobacterium* and *Lactobacilli* in crude model. *: P < 0.05, **: P < 0.01, ***: P < 0.001. Top 8 most abundant infant gut microbiota from *Bifidobacterium* and *Lactobacilli* were explored. Only individual HMOs concentration was included. The relative abundance of infant gut microbiota was transformed in the form of lg (X+1). 2'-FL: 2'-fucosyllactose, 3-FL: 3-fucosyllactose, DFL: difucosyllactose, LNFP-I: lacto-N-fucopentaose I, LNFP-II: lacto-N-fucopentaose II, LNFP-III: lacto-N-fucopentaose III,

LNDFH-I: lacto-N-difucohexaose I, LNDFH-II: lacto-N-difucohexaose II, LNnDFH-I: lacto-N-neodifucohexaose I, LNnDFH-II: lacto-N-neodifucohexaose II, MFLNH-I: monofu-cosyl-lacto-N-hexaose I, MFLNH-III: monofucosyl-lacto-N-hexaose III, MFLNnH: monofucosyl-lacto-N-neohexaose, DFpLNnH: difucosyl-para-lacto-N-neohexaose, LNT: lac-to-N-tetraose, LNnT: lacto-N-neotetraose, 3'-SL: 3'-sialyllactose, 6'-SL: 6'-sialyllactose, LSTb: sialyllacto-N-tetraose b, LSTc: sialyllacto-N-tetraose c, DSLNT: disialyllac-to-N-tetraose, 3'-SLNFP-II: 3-sialyl-latco-N-fucopentaose II, 6'-SLNFP-VI: 6-sialyl-latco-N-fucopentaose VI, Sum: total concentration of the HMOs detected.

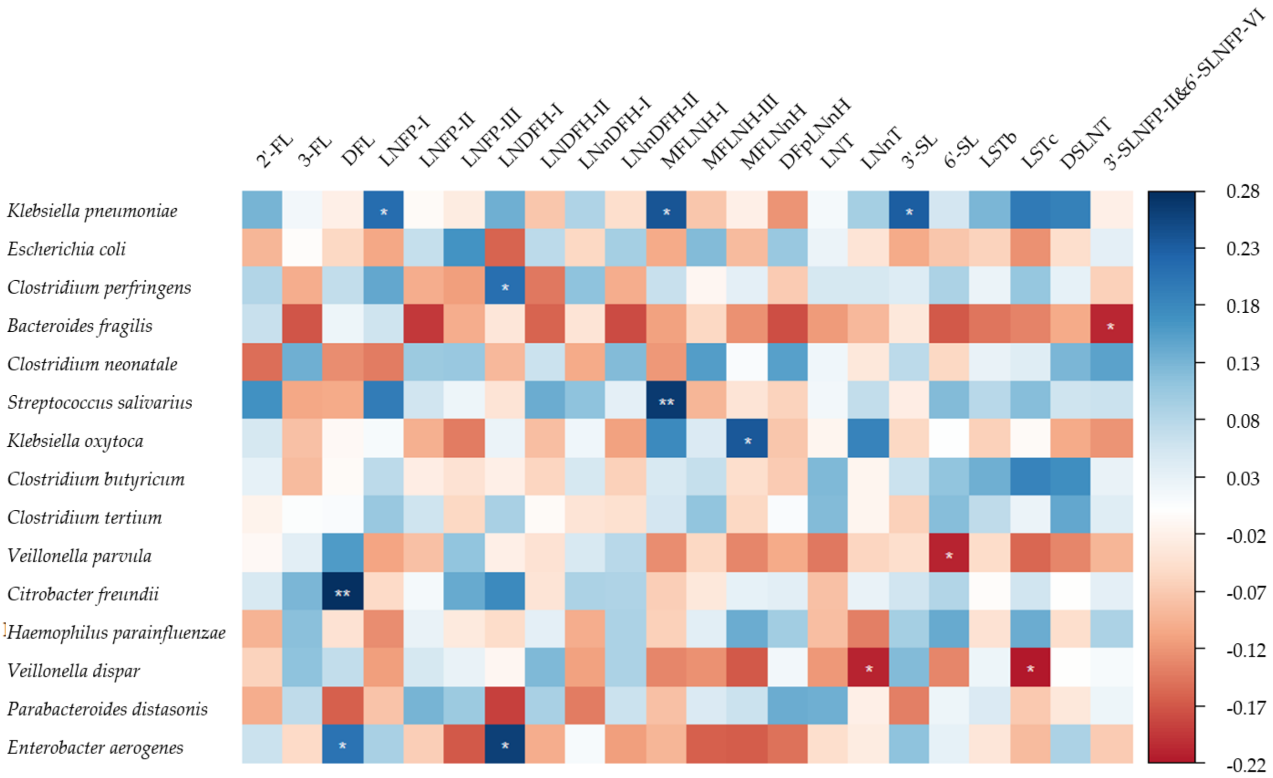

Supplemental Figure S2. Standardised coefficients between individual HMOs concentrations and relative abundance of commensal bacteria. *: P < 0.05, **: P < 0.01, ***: P < 0.001. Top 15 most abundant infant gut microbiota except for *Bifidobacterium* and *Lactobacilli* were explored. Only individual HMOs concentration was included. The relative abundance of infant gut microbiota was transformed in the form of lg (X+1). 2'-FL: 2'-fucosyllactose, 3-FL: 3-fucosyllactose, DFL: difucosyllactose, LNFP-I: lacto-N-fucopentaose I, LNFP-II: lacto-N-fucopentaose II, LNFP-III: lacto-N-fucopentaose III, LNDFH-I: lacto-N-difucohexaose I, LNDFH-II: lacto-N-difucohexaose II, LNnDFH-I: lacto-N-neodifucohexaose I, LNnDFH-II: lacto-N-neodifucohexaose II, MFLNH-I: monofu-cosyl-lacto-N-hexaose I, MFLNH-III: monofucosyl-lacto-N-hexaose III, MFLNnH: monofucosyl-lacto-N-neohexaose, DFpLNnH: difucosyl-para-lacto-N-neohexaose, LNT: lac-to-N-tetraose, LNnT: lacto-N-neotetraose, 3'-SL: 3'-sialyllactose, 6'-SL: 6'-sialyllactose, LSTb: sialyllacto-N-tetraose b, LSTc: sialyllacto-N-tetraose c, DSLNT: disialyllac-to-N-tetraose, 3'-SLNFP-II: 3-sialyl-latco-N-fucopentaose II, 6'-SLNFP-VI: 6-sialyl-latco-N-fucopentaose VI, Sum: total concentration of the HMOs detected.

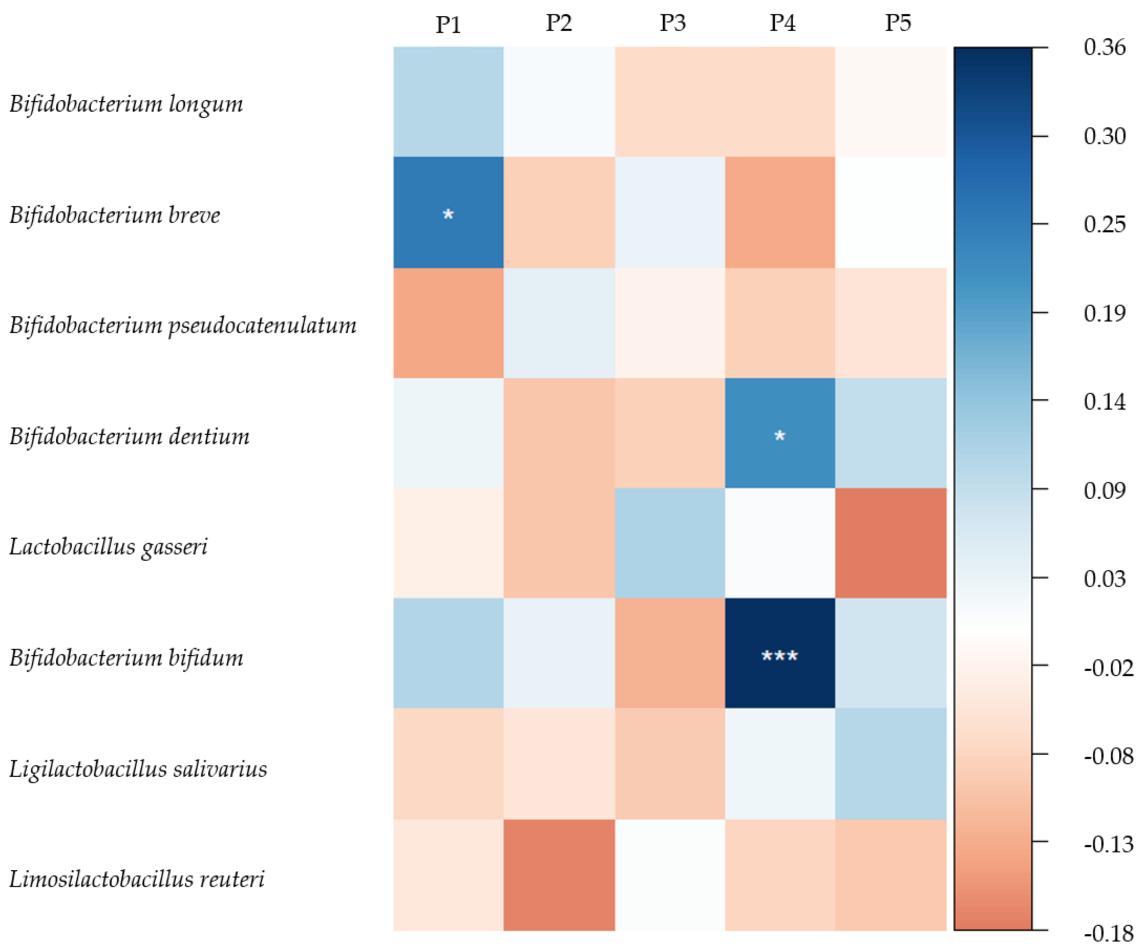

Supplemental Figure S3. Standardised coefficients between HMOs pattern scores and relative abundance of *Bifidobacterium* and *Lactobacilli* in crude model. *: $P < 0.05$, **: $P < 0.01$, ***: $P < 0.001$. Top 8 most abundant infant gut microbiota from *Bifidobacterium* and *Lactobacilli* were explored. Only HMOs pattern score was included. The relative abundance of infant gut microbiota was transformed in the form of lg (X+1).

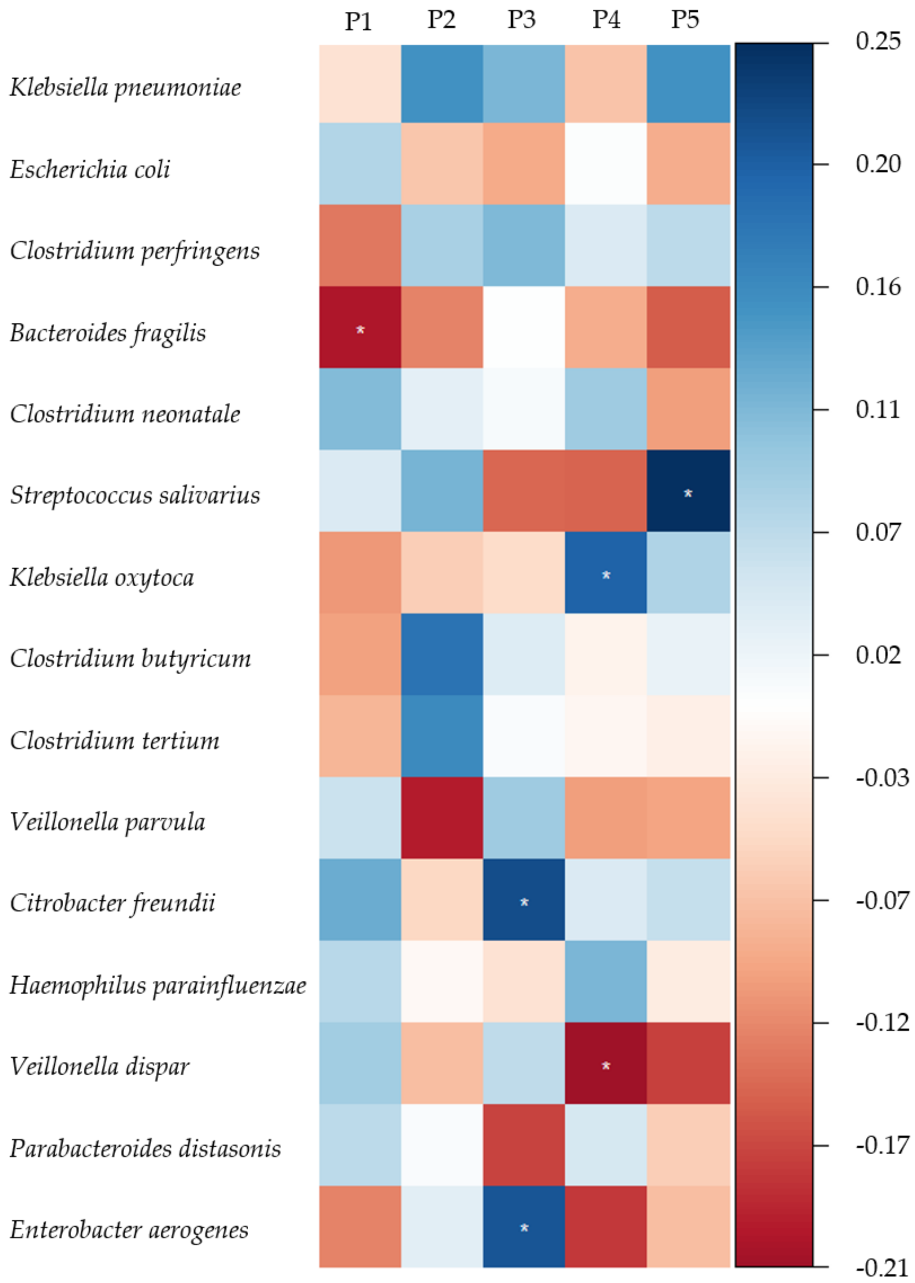

Supplemental Figure S4. Standardised coefficients between HMOs pattern scores and relative abundance of commensal bacteria. *: $P < 0.05$, **: $P < 0.01$, ***: $P < 0.001$. Top 15 most abundant infant gut microbiota except for *Bifidobacterium* and *Lactobacilli* were explored. Only HMOs pattern score was included. The relative abundance of infant gut microbiota was transformed in the form of lg (X+1).
